# Mediators of Physical Activity Protection against ROS-Linked Skeletal Muscle Damage

**DOI:** 10.3390/ijms20123024

**Published:** 2019-06-20

**Authors:** Sergio Di Meo, Gaetana Napolitano, Paola Venditti

**Affiliations:** 1 Dipartimento di Biologia, Università di Napoli Federico II, Complesso Universitario Monte Sant’Angelo, Via Cinthia, I-80126 Napoli, Italy; serdimeo@unina.it; 2 Dipartimento di Scienze e Tecnologie, Università degli Studi di Napoli Parthenope, via Acton n. 38-I-80133 Napoli, Italy; gaetana.napolitano@uniparthenope.it

**Keywords:** insulin resistance, cancer, cardiovascular disease, neurodegenerative disorders, exercise, mitochondria, oxidative stress, PGC-1, Nrf2, UCPs

## Abstract

Unaccustomed and/or exhaustive exercise generates excessive free radicals and reactive oxygen and nitrogen species leading to muscle oxidative stress-related damage and impaired contractility. Conversely, a moderate level of free radicals induces the body’s adaptive responses. Thus, a low oxidant level in resting muscle is essential for normal force production, and the production of oxidants during each session of physical training increases the body’s antioxidant defenses. Mitochondria, NADPH oxidases and xanthine oxidases have been identified as sources of free radicals during muscle contraction, but the exact mechanisms underlying exercise-induced harmful or beneficial effects yet remain elusive. However, it is clear that redox signaling influences numerous transcriptional activators, which regulate the expression of genes involved in changes in muscle phenotype. The mitogen-activated protein kinase family is one of the main links between cellular oxidant levels and skeletal muscle adaptation. The family components phosphorylate and modulate the activities of hundreds of substrates, including transcription factors involved in cell response to oxidative stress elicited by exercise in skeletal muscle. To elucidate the complex role of ROS in exercise, here we reviewed the literature dealing on sources of ROS production and concerning the most important redox signaling pathways, including MAPKs that are involved in the responses to acute and chronic exercise in the muscle, particularly those involved in the induction of antioxidant enzymes.

## 1. Introduction

For several years the practice of physical activity has expanded in scope from competitive sports to disease prevention and health promotion. Therefore, physical activity has been widely recognized as a means for the primary prevention of chronic diseases as well as for patient treatment and rehabilitation [1]. Furthermore, regular physical activity (training) has beneficial effects on people’s health and well-being.

The results of numerous studies have shown that regular physical activity reduces risk of several diseases including cardiovascular diseases, type 2 diabetes (T2DM), some types of cancer, osteoporosis, fall-related injuries, depression, and obesity [1,2,3].

Despite these clear benefits, little is known about the adaptive mechanisms involved in the protection offered by exercise even though to date accumulating evidence has allowed establishing that the production of free radicals represents a potential link between exercise and protection against diseases. 

Currently, free radicals are recognized to play a crucial role in the regulation of critical physiological processes at both the cellular and system level, and be involved, as causal factors, in the development of pathological conditions. The regulatory role of free radicals is a relatively recent discovery, because for several decades they were thought to cause exclusively damaging effects and were gradually implicated in various pathologies, including cardiovascular disease, diabetes, rheumatoid arthritis, cancer, and neurodegenerative disorders [4]. 

The double role played by oxidants in living systems seems to be dependent on the extent of their production. Indeed, if produced in a massive extent, oxidants cause oxidative damage and tissue dysfunction whereas, when moderately produced, they serve as molecular signals activating adaptive responses that are useful for the organism. 

A paradigmatic example is provided just by the exercise. Indeed, a single session of strenuous or prolonged exercise leads to the production of high amounts of radicals and other reactive oxygen species (ROS), which cause tissue damage and dysfunction. Conversely, the single sessions of a training program produce low amounts of ROS, which can induce adaptive responses beneficial for the organism [5].

Interestingly, the incidence of some ROS associated diseases, among which T2DM, rheumatic arthritis, heart disfunctions, Alzheimer and Parkinson diseases, is reduced by the execution of regular physical activity [1,6]. 

The balance of free radicals inside the skeletal muscle is very important, particularly in the context of exercise and sport as the main adaptations occur in the trained skeletal muscle, which can differ with the type of exercise but seem to be nevertheless dependent on ROS production. Thus, aerobic physical activity induces skeletal muscle adaptive responses [1] able to determine an increased resistance to conditions, among which prolonged or strenuous exercise, in which ROS production increase [7,8,9]. Conversely, heavy resistance exercise determines hypertrophy and increased strength production but does not change biochemical characteristics of muscle cells. On the other hand, metabolism of glucose and lipids in skeletal muscles during the resting state and insulin action in insulin-resistant individuals are improved by both aerobic [10] and resistance [11] exercises in skeletal muscle leading to decreased conversion rates to overt diabetes.

In recent times, much progress has been made in understanding the mechanisms underlying the adaptations evoked in skeletal muscle. However, other studies are needed to understand what factors lead ROS to become signal and/or stress agents and the molecular mechanisms through which ROS directly interact with critical signaling molecules to initiate signaling in skeletal muscle. This review, after examining the link between physical activity and ROS production, focuses on signaling pathways, such as MAPKS and transcription factors and cofactors, through which ROS produced in the regular physical activity elicit the adaptive responses implicated in increased antioxidant defenses effectiveness and mitochondrial content of skeletal muscle. 

## 2. Reactive Oxygen and Nitrogen Species

Until about the mid-20th century free radicals, whose existence in chemical systems had been demonstrated by Gomberg’s work [12], were still believed as too reactive species to exist in vivo [13]. Subsequently, when free radical existence in biological systems was recognized, they were thought to cause exclusively damaging effects and to be involved in the development of pathological conditions. In particular, ROS were thought to be involved in the general aging process and in many age-associated diseases [14]. 

This view was mainly supported by the finding that ROS, reacting with most biological macromolecules, cause their oxidative modification, which can result in the loss of their function [15]. In fact, ROS include both highly reactive species, such as the hydroxyl radical (^•^OH), which reacts soon after its formation, and less reactive species, among which are superoxide (O_2_^•−^) and hydrogen peroxide (H_2_O_2_) [16]. Similarly, other reactive species containing nitrogen, named reactive nitrogen species (RNS), include both species not very reactive, such as nitric oxide (NO^•^), and species very reactive such as the peroxynitrite (ONOO^−^), which originates from NO^•^ and is a very strong oxidant for biomolecules and can also undergo decomposition releasing small amounts of ^•^OH [17].

**Figure 1:**
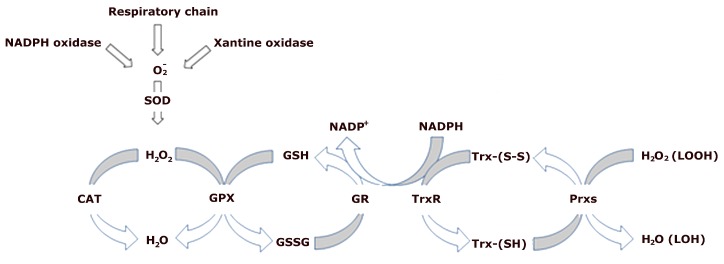
Reactions by which reactive oxygen species (ROS) are produced and removed by antioxidant defense system in skeletal muscle.

Many studies have reported that in normal conditions potentially toxic ROS and RNS are constantly produced at a low level in living systems. Aerobic organisms are equipped with an integrated system of antioxidant defenses to counteract the effects of ROS and RNS [18]. The antioxidant network consists of free radical scavengers of low molecular weight and a composite enzymatic system, which can scavenge free radicals, interrupt chain reactions, remove or repair the damaged components in the cells. Enzymatic antioxidants include a family of metalloenzymes called superoxide dismutases (SODs) [19], which convert O_2_^•−^ to H_2_O_2_, catalase (CAT) an enzyme catalyzing the decomposition of H_2_O_2_ to H_2_O and O_2_ [20], glutathione peroxidases (GPXs) [21], a family of selenoproteins which decomposes H_2_O_2_ using as substrate the reduced glutathione (GSH) which is converted to oxidized glutathione (GSSG), glutathione reductase (GR) which reduces GSSG and restores GSH utilizing NADPH as a source of reducing equivalents [22] (Figure 1). In the cells the redox homeostasis is maintained other than the redox couple GSH/GSSG also by the thioredoxin proteins (Trxs), which are involved in the reduction of protein disulfide [23] and are regenerated by thioredoxin reductase (TrxR) and NADPH [24]. Trxs also collaborate with peroxiredoxins (Prxs) in the hydroperoxide removal as Prxs reduce both hydrogen peroxides and lipid hydroperoxides to water and alcohol with the help of the proteins containing thiol such as Trxs [25]. It is worth noting that Trxs, GPX, and SOD have also been recognized as potential systems able to remove RNS [26].

Normally, the antioxidant system rapidly removes ROS and RNS before they cause cellular dysfunction and eventual cell death. However, in the living systems generation slightly overcome the capacity of the antioxidant defense system to neutralize ROS, therefore a modest level of oxidative damage is always present. Probably, ROS are not all eliminated because they perform important roles, so that the challenge for the survival process was to evolve antioxidant defenses that allow such roles while minimizing damage. 

However, when a greater imbalance occurs in favor of the ROS, oxidative stress ensues [27] characterized by widespread tissue damage, to which cells can adapt sometimes by upregulating the antioxidant system.

## 3. Exercise Induced Oxidative Damage

It is long known that physical activity promotes well-being and that in inactive subjects there is an increased incidence of several chronic diseases including obesity, diabetes, hypertension, osteoporosis and mood problems.

The observation that exercise, long-lasting in trained and of short term in non-trained subjects, induces damage seemed at odd with the idea of its beneficial effects. The damage was mainly observed in exercise in which the eccentric contractions were prevalent, and it included structural and functional alterations not only in skeletal muscles but also in other tissues [28,29,30,31]. 

ROS implication in tissue damage induced by acute exercise was reported as early as the late 1970s [32]. Some years later, Davies and collaborators [33], using electron spin resonance (ESR) spectroscopy method proved first that free radical signals were intensified in rat muscle after a bout of exhaustive running. These results were subsequently confirmed by Jackson et al. [34]. It was later observed that contracting muscles also produce NO^•^ and other RNS [35]. 

Since these early observations, many studies have confirmed that muscular exercise promotes the production of both ROS and RNS in skeletal muscle fibers. Jenkins et al. [36] found that the tert-butyl-hydroperoxide induced chemiluminescence, a marker of ROS production, was increased by an exhaustive run in the hindlimb muscles of rats. Furthermore, it was found that ROS production, measured using the intracellular probe 2′,7′-dochlorofluorescein, increased in rat diaphragm muscle during contraction [37], in vastus lateralis after exhaustive exercise [38], and in single mature skeletal muscle fiber [39].

Important advances have also been made about to identification (principally) and quantification of reactive species. Indeed, a series of studies demonstrated that contracting skeletal muscle transiently overproduces parent reactive species, such as O_2_^•−^ and NO^•^, and secondary reactive species, such as H_2_O_2_, ^•^OH, ONOO^−^, and lipid-derived oxygen (O_2_)-centered alkoxyl radicals [39,40,41,42,43]. However, the direct mechanisms and sources of ROS and RNS production during exercise remain uncertain and they are likely to differ depending on the type of activity.

In the cell, O_2_^•−^ is generated by the addition of a single electron to ground state oxygen in several sites including plasmalemma, cytosol, peroxisomes, mitochondria and endoplasmic reticulum [5]. 

It has been found that the mitochondria, the NADPH oxidase (NOX), and the enzyme xanthine oxidase (XO) are the main endogenous sources of ROS in skeletal muscle [44]. In mitochondria, superoxide production verifies mainly at complexes I and III of the electron transport chain [45], and it has often been assumed that it is the primary cell source of ROS in physiological and pathological conditions [46]. However, there is no convincing evidence that mitochondria are the main cellular source of ROS in contracting muscle fibers [47]. Conversely, using confocal microscopy with specific fluorescent probes it was observed that muscle contraction increases O_2_^•−^ in cytosol and subsequently in mitochondria. This observation suggests that a ROS generator different from mitochondria could be the potential primary source of ROS production during muscle contraction [48]. Furthermore, the idea that mitochondria are the main source of ROS during the muscle contraction is also theoretically inconsistent. In fact, when muscle contraction begins mitochondrial respiration enters State 3 (active respiration). Since the reduction degree of the autoxidizable carriers, by which mitochondrial ROS production depends, decreases in State 3 [49], it is foreseeable that the rate of O_2_^•−^ production also decreases during the muscle contraction. 

Nevertheless, measurements of ROS release by muscle mitochondria isolated from exercised animals suggest that the rate of mitochondrial ROS release is increased by aerobic exercise. For example, ROS release by mitochondria isolated from rat exercised to swim until exhaustion increased during both State 4 and State 3 respiration [50,51]. This increase was accompanied by alterations in mitochondrial functionality as evidenced by enhanced State 4 and decreased State 3 respiration [50]. Thus, it is possible to hypothesize that during exercise a source other than mitochondria initially produces the ROS, which then damage the mitochondria altering their functionality and increasing their ROS release.

NOX, located within the sarcoplasmic reticulum, transverse tubules and sarcolemma, is considered a key ROS generator during muscle contractions. Indeed, both at rest and during contractile activity it appears to contribute more than mitochondria to cytosolic O_2_^•−^ in skeletal muscle [52].

On the other hand, evidence also indicates that XO produces superoxide in the cytosol of contracting rat skeletal muscles [53] even if it has also been reported that the muscle cells do not contain large amounts of the enzyme [54]. However, this enzyme is present in associated endothelial cells and might contribute to exercise-induced muscle damage [55]. However, additional research is required to determine the role played by XO in exercise-induced ROS production in human skeletal muscle.

Long lasting, strenuous exercise also induces oxidative muscle damage via ROS production by phagocytic white blood cells, particularly neutrophils, which infiltrate the muscular tissue [56]. NO^•^ is produced from the conversion of L-arginine into L-citrulline by enzymes known as nitric oxide synthases (NOS) [57] of which three different isoforms have been defined: Type I neuronal (nNOS), type II inducible (iNOS) and type III endothelial (eNOS). Normally, skeletal muscle expresses nNOS and eNOS, whereas iNOS, is induced in response to infection, inflammation, or trauma [58]. Thus, it is well established that isolated skeletal muscle fibers produce low levels of NO^•^ during resting conditions while heavy muscle contraction results in increase of generation of NO^•^ which has many signaling functions but may also have some detrimental effect because of the danger linked to the formation of highly reactive ONOO^-^ [42]. Passive stretching of the muscle has also been shown to increase NO^•^ release from rat skeletal muscle in vitro and to increase nNOS expression. Moreover, the use of inhibitors of putative generating pathways [42] and western blotting [43] indicates that contraction induced NO^•^ release is primarily from neuronal NO^•^ synthase enzyme.

## 4. Markers of Exercise-Induced Oxidative Damage

In addition to the ROS formation during exercise having been directly measured, other support exists to the idea that during exercise an increased ROS production verifies. Indeed, it is possible to determine the changes in tissue content of stable molecules arising from the reaction of free radicals with certain biomolecules. In fact, the increase in radical production often results in profound oxidative alterations of various biological substances, including lipids, proteins and nucleic acids, which are commensurate to the increase in free radical production. Thus, the measurement of the content of derivatives of these substances oxidatively damaged has been used to obtain information on ROS production in various physio-pathological conditions. Most commonly measured are the molecules derived from the oxidatively damaged lipids, proteins or DNA or the changes in the levels of antioxidant molecules such as GSH (Table 1). 

Lipid peroxidation has been frequently considered as a marker of exercise-induced oxidative stress thanks to the extreme susceptibility of lipids to ROS and to the stability of lipid peroxidation byproducts. Therefore, different studies exist showing that lipid peroxidation increase in skeletal muscle after acute running [33,59,60,61] and swimming exercise [8,9,50].

In contrast to lipid peroxidation, few data are available on the effects of exercise on protein and DNA oxidation in skeletal muscle. 

Protein oxidative damage generates several byproducts that originate from oxidative modifications of lateral chains of different amino acids. Protein carbonyls represent an irreversible form of protein modification and have been demonstrated to be relatively stable, so that they are considered an adequate marker of protein oxidation [62]. The first study on accumulation of protein-bound carbonyls was published by Reznick et al. [63], who reported that a single bout of exercise caused an increase in protein-bound carbonyl content in the rat skeletal muscle. Similar results were subsequently obtained in rat skeletal muscle subjected to exhaustive exercise [64]. Conversely, other researchers found that protein carbonyl formation in deep vastus lateralis [38] and in both fast and slow muscles [65] was unaffected by exhaustive exercise. More recently, oxidative damage to proteins in homogenates and mitochondria from skeletal muscle has been found after swimming exercise [50].

To establish DNA oxidative damage, the accumulation of 8-hydroxy-deoxyguanosine (8-OHdG) is normally determined. The levels of 8-OHdG in several tissues of dog, including skeletal muscle, showed no significant changes in tissues, except the colon, soon after exercise [66]. Similarly, no significant changes were found in the levels of 8-OHdG in the nuclear DNA of fast and slow muscles of rat because of acute exercise [65].

In contrast, increases in 8-OHdG were found in skeletal muscle from young and old subjects 24 h after a single bout of exercise [67]. It was suggested that the increase in DNA damage was due to a delayed effect of exercise, which results in activation of macrophages and neutrophils and involves massive ROS production. 

Some explanations can be provided for the lack of 8-OHdG increase soon after the end of the exercise. First, it is likely that most of the ROS generated by various cellular sources during the exercise are intercepted by cytosolic antioxidants before they reach the nuclear DNA. Second, the DNA in the nucleus is protected by the histone proteins, which render the nuclear DNA less susceptible to ROS activity [68]. Finally, a specific enzyme, the 8-oxoguanine DNA glycosylase/lyase [69], is activated and rapidly repairs oxidatively damaged DNA [70]. 

GSH, a ubiquitous tripeptide thiol, is one of the most important scavengers of ROS, and its ratio with GSSG may be used as a marker of oxidative stress, because GSH is oxidized to GSSG, and the GSH/GSSG ratio decreases under oxidative conditions. Several studies have reported a decrease in muscle GSH/GSSG ratio in response to exercise. An early study by Lew and coauthors [71] showed that exhaustive exercise causes significant increases in GSSG and ratio between GSSG and total glutathione (GSH + GSSG) in rat skeletal muscle. Subsequently it was shown that GSSG was elevated to as high as 160% of the resting levels, whereas GSH/GSSG ratio fell significantly after an exhaustive bout of exercise [72]. More recent research has also shown that 6 h of swimming exercise reduces muscle GSH level and GSH/GSSG ratio [50].

Interestingly, like aerobic exercise, anaerobic exercise of enough intensity and duration increases oxidative modification of proteins, nucleic acids, and lipids [73]. However, aerobic exercise-induced ROS release and consequent oxidative damage depends on mitochondrial electron transport chain and on the enzyme NADPH oxidase, which is localized in the sarcoplasmic reticulum, the transverse tubules and on the muscle plasma membrane [74]. Conversely, the ROS production found during and after anaerobic physical activity can be due to other systems, among which xanthine oxidase [75].

In the whole, despite some disagreeing results, there seems to be little doubt that acute exercise results in both enhanced production of reactive species and oxidative damage to components of muscular cells. 

An important consequence of the involvement of the free radicals in tissue damage caused by acute exercise is the possibility to reduce the radical effects by supplementation with antioxidants such as vitamins C and/or E, carotenoids, GSH or its precursor the N-acetylcysteine. It has been shown that antioxidant supplementation protects against the deleterious effects of intense exercise [76,77,78]. The involvement of free radical in the exercise-induced oxidative damage and the protective effect of antioxidants are further demonstrated by observation that low levels of vitamin E are associated with a high exercise induced lipid peroxidation [33].

## 5. Muscle Adaptations Induced by Training

Skeletal muscle is particularly responsive to training which induces adaptations such as potentiation of antioxidant system, increased mitochondrial content, increased sensitivity to insulin, ameliorating the muscle function and protecting against the onset of metabolic disorders [79].

An important concept developed over the past decade is that the responses to training are likely the result of the acute but cumulative effects of the responses to single exercise bouts [80]. Thus, each bout of exercise initiates acute and transient changes in gene transcription which are reinforced by repeated exercise stimuli, leading to altered, chronic expression of a variety of nuclear and mitochondrial DNA (mtDNA) gene products, that ultimately form the basis of skeletal muscle training adaptation and improvements in exercise capacity [81]. 

However, skeletal muscle responds to exercise in a training specific manner. Classically, training was distinguished in “endurance training” and “strength training” also referred as “resistance training”. Endurance exercise (e.g., running, swimming, cycling) is generally characterized by high-frequency, long duration, and development of a relatively low force. Resistance exercise (e.g., weight lifting) is, in general, characterized by low frequency, short duration, and development of a relatively high force. These two training modalities represent the extremes of a continuum of exercise protocols of countless options that differ in terms of intensity, duration, frequency, and mode of contraction as well as any combination of these.

Endurance training enhances the muscle aerobic metabolism capacities but do not induce increases in muscle mass or capacity to develop strength. Indeed, training to endurance determines in skeletal muscles a transformation of fiber-type, increases the mitochondrial mass, the production of new blood vessels and other adaptations [82]. Muscle blood vessels increase is a necessary adaptation to the increased mitochondrial oxygen requests [83]. Mitochondrial density increases rapidly in muscle particularly when subjects are previously untrained. The increase in mitochondrial compartment is accompanied by enhancement in the content of the enzymes of both Krebs cycle and oxidative phosphorylation among which succinate dehydrogenase (SDH), citrate synthase (CS), and cytochrome *c* oxidase (COX) [84]. Due to these adaptations, and of the increased capillarization, in the endurance trained muscle oxidative capacities are greatly enhanced. Conversely, endurance exercise does not change the cross-section area of the fibers unless the muscle was preceded by immobilization or underuse [85].

The adaptations elicited by the endurance-type exercise increase the resistance to exercises of intensities that in the untrained state can be performed for shorter period. 

Strength training induces muscle cells hypertrophy and increase strength production but does not affect biochemical composition. Classic strength training protocols predominantly impact on muscle and muscle fiber cross-sectional area. It is important to realize that, in terms of functional changes, significant strength gains can be obtained by changes in the nervous control of the muscle mainly at the onset of training session [86]. At the beginning, the functional adjustment can be obtained with low level of structural changes. Continuing the training of strength, the cross-sectional area increases, and this is more evident at the origin and insertion of the muscle [87]. 

It was initially hypothesized that the increase in the cross-section area was due to the expansion of the preexisting cells and not to the cell proliferation. Subsequently, it was shown that such a growth, was dependent on the enhanced content of myofibrils, and that the net increase in cross-section area was mainly due to the increase in the fast fibers of the type IIa and IIX in man [88]. However, evidence is now available that, in several animal species, eccentric strength training, during which muscle exerts force while lengthening, is capable of muscle hyperplasia with neoformation of muscle fibers even though muscle growth depends largely on fiber hypertrophy [89]. The expression of the heavy chain of myosin is changed by strength training in an extension and direction that apparently depends on the characteristics of the protocol of exercise. 

In older adults particularly salutary is resistance training thanks to its capacity to reduce the sarcopenia that verifies with age [90]. Resistance training is advisable for all healthy adults for its beneficial effects in reducing blood pressure [91] and cardiovascular disease risk [92]. 

Early works suggested that strength training only marginally changes mitochondria and capillarization in muscle [93]. Indeed, mitochondrial volumes and capillary densities were found to be low in strength-trained human muscles; muscle metabolism remained dominantly carbohydrate-dependent such that the relative content of cytoplasm containing glycogen was increased [94].

However, more recent works indicate that strength training results in effects like those elicited by endurance training. Indeed, it can improve insulin action and glucose metabolism [11] and stimulate mitochondrial biogenesis [95]. Moreover, recent researches have challenged the view that endurance and strength training are distinct exercise modalities, which increase mitochondrial density [96] and myofibrillar units [88] of skeletal muscle, respectively. It was found that in lean sedentary adults both 10 weeks resistance training or aerobic training enhanced mitochondrial respiration in the skeletal muscle, and that the oxidative capacity increase was dependent on qualitative changes in mitochondria not being the mitochondrial density substantially modified [95]. This suggests that mitochondrial biogenesis is stimulated by both training modalities, although it is likely the two training modalities do not achieve the same outcome by identical mechanisms. 

A subsequent study also showed that a long period (nine months) of resistance and endurance training induce muscle mitochondrial proliferation and that the combination of both training modalities induces a more marked reduction of oxidative damage to lipids and carbohydrates and a greater increase in mitochondria content and mitochondrial enzyme activities, suggesting that the two modes of training together are healthier by protecting against T2DM [97]. 

Interestingly, study performed on elderly muscle, which showed large energetic, but smaller structural, adaptations, demonstrated that only resistance training induced a rise in mitochondrial volume density and muscle size [98].

## 6. Mechanisms of Muscle Adaptive Responses to Training

Accumulating evidence has induced to think that the dual role of ROS in animal organisms can be responsible for the contrasting effects of acute and chronic exercise. Indeed, it is now well established that ROS can damage proteins, nucleic acids and membrane phospholipids leading to cellular dysfunction, but they also have essential physiological functions in the cells acting as signals for the regulation of transduction, proliferation and transcription [99]. Therefore, the different roles of ROS in training as signaling molecules for the induction of tissue adaptation, and in acute exercise as damaging molecules, can depend on differences in the extent and temporal pattern of ROS generation. The low levels of ROS produced intermittently for a short period of time during a training protocol program, activate intracellular signaling ways that promote cellular adaptations leading to increased capacities against subsequent stresses. Conversely, moderate levels of ROS generation for a long period of time, or high generation due to high intensity exercise, induces structural and functional damage. 

In the past years, evidence has been obtained that during each session of a training program the low level of ROS regulates signaling cellular pathways that result in the induction of the training induced adaptations that are healthy for the organism [5]. 

In the subsequent parts of this review we will examine the literature concerning our current knowledge about some potential signaling pathways that link ROS to the remodeling that occurs in skeletal muscle during exercise training. 

## 7. Muscle Performance

It was long accepted that the idea that training to physical exercise led the animals or human to successfully endure exercise loads of different intensities, types and durations. Training shows to be able to stem the homeostasis disturbance during an exercise bout, allowing the animals or humans to bear physical work for longer time before fatigue appears [100], but the factors that condition the physical performance are controversial. 

## 8. ROS and Muscle Performance

In the past years, evidence was obtained that ROS affect the muscle capacity to generate force, being that the low levels of ROS in the resting (i.e., unfatigued) state is necessary for normal force production [101]. Therefore, in the muscle the excessive scavenging of ROS by the antioxidant is linked to reduced force generation [101,102,103], whereas a low ROS production increases force generation [104]. On the other hand, the ROS capacity to increase muscle force production is reversed when ROS levels are higher and force production is reduced with increased time of exposition and dose of ROS [104]. These results led to propose the existence of an ideal redox state in which the conditions for the force production by the muscle are optimal and that the removal from such an ideal state leads to reduced force production [104].

Endogenous production of NO^•^ can also modulate skeletal muscle force production. Indeed, studies using excised bundles of muscle fibers reveal that force production during submaximal tetanic contractions is depressed by NO^•^ donors and increased by NOS inhibitors and NO^•^ scavengers [74]. Conversely, a consensus of literature does not exist to support the notion that NO^•^ production promotes muscular fatigue [74]. The evidence that sports performance is impaired by redox imbalance through various mechanisms that compromise the structure and function of the muscle cells, suggested that ROS contribute to muscular fatigue during prolonged exercise. The process involved in exercise-induced muscle fatigue depends on several factors [105,106] and the specific causes of muscle fatigue vary depending on the type of exercise that produces it [107]. For example, the main factors contributing to fatigue during high-intensity contractions that take place during resistance exercises and that contributing to fatigue during low intensity exercise with continued contraction, are different. However, there is evidence indicating that free radical production in skeletal muscles contributes to fatigue during various types of exercise. 

Indeed, by studying the contribution of oxidants to muscle fatigue using a variety of animal model evidence of a relationship between endurance and free radical generation was obtained. Several studies have demonstrated that antioxidant treatment can delay the fatigue, but other studies have shown that antioxidants are able to reduce the levels of oxidative stress markers but not the onset of fatigue, mainly in humans [108]. However, most human studies have been performed on athletes or trained subjects, in whom the antioxidant supplementation can have harmful effects that hinder the adaptive processes stimulated by ROS (see below). Indeed, endurance to isokinetic cycle exercise was increased in healthy untrained volunteers by diet supplementation with a whey-based cysteine donor [109]. Furthermore, the administration of vitamin E, which is known to reduce the exercise-induced oxidative damage [110], prolonged the endurance to physical exercise in mice [111]. Novelli et al. [112] also observed that directly administered GSH to mice resulted in an increase in swimming endurance. The most effective substance in inhibiting fatigue was *N*-acetylcysteine (NAC) [113], a nonspecific antioxidant and reduced thiol donor that is a precursor and upregulator of the synthesis of GSH [114]. The evidence that muscle performance is most consistently improved by antioxidants that oppose thiol oxidation leads to think that such antioxidants delay fatigue by helping to maintain thiol groups of myofibrillar proteins in a reduced state.

## 9. Training and Muscle Fatigue

In contrast to acute exercise, training does not increase muscle oxidative damage as demonstrated by the finding that levels of malondialdehyde (MDA), a product of lipid peroxidation, of white muscle are not modified whereas those of red muscle are decreased in rats trained to run [60]. Furthermore, levels of MDA and of lipid hydroperoxides, another product of lipid peroxidation, are not modified in gastrocnemius of young [8] and adult [9] rats trained to swim. 

Training also exerts a protective effect against oxidative damage elicited by acute exercise since it prevents the appearance of some signs of exercise-induced free radical generation, such as the increases in muscle lipid oxidation normally elicited by acute run exercise [60]. In contrast, exhaustive swimming exercise gives rise to tissue damage irrespective of the trained state, as documented by similar levels of muscle lipid peroxidation and loss of SR and ER integrity found in exhausted trained and untrained rats [8]. However, exercise endurance capacity is greatly increased in trained rats indicating that lipid peroxidation and tissue damage are strongly slowed down. 

## 10. Antioxidants Enzymes

An explanation for these training effects was provided by the finding that muscle activities of the key antioxidant enzymes were modified by chronic exercise protocols [115]. The results obtained in subsequent studies were somewhat variable, but in general they confirmed the idea that exercise training promotes an increase in key antioxidant enzymes in skeletal muscle (Table 2). Thus, glutathione peroxidase (GPX) activity was increased, whereas SOD was unmodified and CAT activity was decreased in skeletal muscle of rats trained to run [116]. GPX and GR activities were increased by swimming training in gastrocnemius muscles of young (four months) [8,117] and adult (12 months) rats [9]. Training increased total antioxidant capacity irrespective of age [8,9], but its effects on antioxidant enzymes were dependent on age. Indeed, training increased GPX and SOD activities in young rats, decreased GR and CAT activities in adult rats and CAT activity in old rats. Thus, it was suggested that exercise training, although increasing selective antioxidant enzymes in young rats, does not offer protection against oxidative stress in the senescent muscle [118]. This view was confirmed by the observation that run training increased the activities of CAT, GPX, and MnSOD, and did not modify CuZnSOD activity in young rats, whereas did not modify the activities of CAT, GPX and CuZnSOD in the soleus muscle and decreased the activity of MnSOD in aged rats, [119]. Moreover, swimming training enhanced the activity of GPX and CuZnSOD, but not that of MnSOD, in young mice, while it did not modify enzyme activities in old mice [120].

The magnitude of adaptation was in part dependent upon exercise intensity, so that higher training intensities induced greater changes in the antioxidant defense [121]. Moreover, the changes induced by training in muscle antioxidant enzymes were muscle fiber-specific because GPX and SOD activities increased in vastus lateralis, whereas GR activity declined and those of GPX and SOD remained unchanged in soleus [122].

Antioxidant adaptation is also dependent upon exercise duration. Indeed, GPX and GR activities, which were unchanged after the first six-week training period, increased after the 7th week of training, whereas SOD activities were unchanged after both training periods [54].

It is worth noting that chronic resistance training may provide a protective effect like aerobic exercise on redox homeostasis. Indeed, it was reported that resistance training reduces serum lipid peroxidation providing protection against oxidizing agents in vitro and against oxidative damage generated by aerobic exercise [123], perhaps mediated by improvements in the thiol portion of the antioxidant defense. Furthermore, resistance training reduces muscle DNA oxidative damage [124] and increases antioxidant defense in older adults [125]. Indeed, training results in a significant increase in CuZnSOD and CAT but not MnSOD activity in vastus lateralis muscle [125].

Several experimental evidences suggest that the training-linked adaptations are induced by changes in gene expression with upregulation of both mRNA levels and protein expression. It must be underlined that the available data are few and debatable. For example, CuZnSOD activity and mRNA abundance were found to be higher in vastus lateralis (VL) muscle of trained compared to sedentary rats. However, the CuZnSOD protein content was not altered in the muscle, but increased in its superficial portion (type 2b). Moreover, the MnSOD protein content was higher in trained rats but its activity and mRNA abundance were not affected, whereas the GPX activity was increased without changing its mRNA abundance [126].

Training increased mitochondrial MnSOD activity in the deep portion of VL, and the MnSOD protein content in pooled superficial portion of VL and plantaris muscle [127]. The levels of the mRNA of MnSOD did not change in any muscle. The mRNA level, protein content and activity of CuZnSOD were not changed by training except for an increased protein content in pooled SVL. GPX and CAT activities were increased significantly by training only in the muscle DVL. Therefore, it was suggested that training induces adaptations of the antioxidant enzyme mainly in fibers of the type IIa, probably for the increased free radical production and for the low antioxidant capacity. The different training effects on mRNA, content of the enzyme protein and activity indicate that different cellular signals can affect the pre and post translational regulation of SOD. 

It is worth noting that even acute bouts of exercise were found to be able to increase the activities of antioxidant enzymes, including SOD, CAT, and GPX [72,128,129] and GR [128], in skeletal muscle. The threshold and the greatness of the activation appeared different among enzymes and were fiber-specific because the enzyme activities increased in deep portion of the vastus lateralis but not in soleus [128]. The mechanisms by which antioxidant enzymes could be activated within a relatively short period of time during the exercise were largely unknown even though the fast activation suggested that enzymatic molecules underwent allosteric or covalent modifications. The rapid activation of antioxidant enzyme synthesis by oxidative stress through a transcriptional pathway had been shown in prokaryotes (*Salmonella and Escherichia)* [130], but there was no evidence that a similar mechanism existed in mammalian cells [131].

Subsequent studies showed muscle fiber specific upregulation of superoxide dismutase gene expression in skeletal muscle [132]. Indeed, increases in MnSOD mRNA levels were found in the DVL 0, 1, and 2 h after exercise, whereas MnSOD protein levels were not changed. MnSOD mRNA levels were not modified by exercise in SVL, whereas MnSOD protein levels were increased 10 and 24 h after exercise. CuZnSOD mRNA levels were not changed by exercise in DVL and SVL, whereas the CuZnSOD protein content was increased 48 h after exercise in both muscles. Activities of MnSOD, CuZnSOD and total SOD were not modified by exercise in either muscle [132]. The increases in the CuZnSOD protein content seen post-exercise, without increases in mRNA abundance in both DVL and SVL, suggested a translational mechanism in this SOD isoform [132].

Moreover, more recent studies showed that in monocytes, after individual exercise bouts, target genes of the nuclear transcription factor, peroxisome proliferator activated receptor-γ (PPARγ), were upregulated at the mRNA level up to 3 h after each exercise, and this effect persisted for less than 24 h [133,134]. In contrast, after an eight-week training program, increases in gene expression were observed at the protein level in samples taken 48 h after the previous bout of exercise [134]. 

Such a view was supported by subsequent studies which showed that increase in PPARγ target gene expression observed after single bouts of exercise were similar, but less pronounced, to that seen after eight-week training programs involving at least three exercise bouts per week [135]. Thus, a study using the mass spectrometry coupled with liquid chromatography (LC-MS/MS) to investigate the effect of an acute bout of endurance exercise on protein composition of human vastus lateralis (VL) muscle in endurance trained and untrained individuals, found that training altered the content of 92 structural and mitochondrial proteins. In contrast, a single bout of exercise (3 h) resulted in an alteration of the content of 44 proteins in untrained athletes [136].

These results suggest that the effects of each exercise bout can merge so that, after a training program, a more sustained effect is apparent. It can therefore be concluded that acute exercises, except for the most intense ones, which cause oxidative damage, result in small transient oxidative stresses which, in turn, induce redox-sensitive responses on local and systemic level, thus contributing to training adaptations and systemic health benefits.

## 11. ROS Production

Although it was apparent that training effects on muscle performance were associated to an increase in the effectiveness of antioxidant defense system, it was not known whether such an increase was the only change responsible for the slowdown of the peroxidative processes and the muscle fatigue. In fact, it was conceivable that a delayed onset of fatigue can also be dependent on adaptations involving a decrease in rate of production of reactive oxygen derivatives. Venditti et al. [137] first found that mitochondrial H_2_O_2_ release supported by succinate was lower in swimming trained than in untrained rats both in State 4 and State 3, whereas that supported by pyruvate/malate was lower only in State 4. The decrease in succinate-linked H_2_O_2_ release was such to compensate for the increase in mitochondrial protein content produced by training in the muscle [137]. Although the ROS produced by mitochondria are only a part of those produced in the cell, the above finding indicated that training can reduce their contribution to the intracellular steady state concentration of H_2_O_2_. 

A subsequent study showed that eccentric training also leads to positive adaptations, decreasing mitochondrial H_2_O_2_ release when mitochondria are incubated with either pyruvate/malate or succinate [138]. It has also been shown that training decreases mitochondrial H_2_O_2_ release in healthy and diabetic subjects [139]. A recent research has shown that immobilization increases H_2_O_2_ emission, while subsequent aerobic training (supervised bicycle training) reverses these effects in young and older men [140]. A more recent study has shown that training decreases H_2_O_2_ production measured in freshly permeabilized soleus muscle in the presence of substrates of he ttricarboxylic acid cycle [141]. 

Surprisingly, another research has shown that training increases mitochondrial ROS production in old subjects with both normal and impaired glucose tolerance [142]. 

It remains unclear whether resistance training affects the mitochondrial ROS production in older adults, because a recent report [143] indicates that resistance training does not induce significant changes in mitochondrial ROS production in vastus lateralis muscle from older adults. 

Although these in vitro models may not correspond to conditions in vivo, overall, the reported results seem to indicate a potential training-induced decrease for H_2_O_2_ release. However, the causes of the reduction of the mitochondrial ROS release induced by training in skeletal muscle are not well understood. One possibility is that the decrease in H_2_O_2_ release elicited by training is not due to a decrease in ROS production, but to a greater capacity of mitochondria to scavenge superoxide radical or hydrogen peroxide, thus limiting the generation of highly reactive hydroxyl radicals. However, the observation that regularly performed moderate exercise does not modify the total antioxidant capacity of mitochondria indicates that the reduction in mitochondrial H_2_O_2_ release is not due to a greater capacity of mitochondria to scavenge reduced oxygen intermediates [137]. This view is supported by the observation that the activities of mitochondrial antioxidant enzymes (MnSOD, CuZnSOD, GPX, GR, and CAT) are not affected by training [138].

Another possibility is that the chronic exercise leads to changes in factors, which can influence mitochondrial free radical production, such as mitochondrial membrane potential. In fact, evidence is available that training produces a drop in mitochondrial membrane potential, although the causes of such a drop remain still unknown [137]. In theory, the drop in mitochondrial membrane potential and, therefore, the decrease in H_2_O_2_ production could be due to increased uncoupling of the inner mitochondrial membrane. Such an uncoupling can be dependent on the levels of members of the mitochondrial transporters family, mitochondrial uncoupling proteins (UCPs), which contribute to energy dissipation as heat by uncoupling respiratory chain from ATP synthesis [144,145]. Of the five mammalian forms of UCPs, UCP4 and UCP5 are principally neuronally expressed [146]. The best characterized of these proteins, UCP1, is expressed exclusively in brown adipose tissue (BAT) [147], UCP2 is expressed in a large spectrum of tissues including skeletal and cardiac muscle, and UCP3 is mainly expressed in BAT and skeletal muscle [148] in which it is involved in decreasing ROS production [149]. 

Since peroxisome proliferator-activated receptor γ coactivator 1α (PGC-1α) can regulate the mRNA expression of Ucp2 and Ucp3 in the muscle cell culture [150], it was suggested that the increased PGC-1α expression induced by training could increase the uncoupling capacity of skeletal muscle mitochondria and thus decrease their ROS production by [151]. However, such an idea is not supported by experimental evidence since increases in UCP3 protein content in rat muscle have been found only after acute exercise [152] and short-term training (10 days) [153]. 

Rather, it has been hypothesized that the decreased ROS production observed with eccentric exercise is due to a mild uncoupling caused by an increase in the ratio between polyunsaturated and saturated fatty acids, and a decrease in the content in arachidonic acid and plasmalogens in mitochondrial membrane [138].

It is worth noting that the possibility that exercise training can lead to a decrease in skeletal muscle ROS production reducing the activity of other cellular sources has not been investigated. Only recently, evidence has been obtained that decrease in ROS production induced by aerobic exercise training in skeletal muscle is associated with reduced muscle NADPH oxidase activity [154].

## 12. Factors Regulating Protein Expression

Although the effects of aerobic exercise on antioxidant capabilities in skeletal muscle have been well described, the regulatory mechanisms underlying this adaptation are complex and incompletely understood. 

In the 90s of the last century accumulating data indicated that cells exposed to ROS responded by inducing or repressing a wide variety of genes [155]. These effects seemed to be due to changes in the intracellular redox balance that influenced multiple signaling pathways leading to a modulation of the expression of some genes [156,157,158]. Several genes that could be differentially regulated by oxidative stress were characterized and included early response genes, genes for enzymes involved in antioxidant protection, and genes for specific stress and heat shock proteins (HSPs) [156,157,158]. 

At present, a complete answer to the question as to how changes in the redox status of muscle fibers regulate signaling pathways and gene expression is not yet available. However, it is known that redox signaling can affect numerous transcriptional activators leading to altered gene expression and modified muscle phenotype. An important mechanism by which redox signaling controls gene expression is the modulation of the phosphorylation state of transcriptional activating factors due to ROS ability to control the activities of many kinases and phosphatases [159,160].

Eukaryotic cells possess many families of kinases, but the family of mitogen-activated protein kinases (MAPKs) represents the main link between cell ROS levels and skeletal muscle adaptive responses.

### 12.1. MAPK

MAPK family is one of the main kinase families that are involved in the conversion of cell signals into cellular responses. MAPKs contribute to the regulation of life-and-death decisions taken in response to several stress signals including ROS [161]. The control exercised by MAPKs on a wide variety of pathways of cellular signaling is obtained through phosphorylation-mediated activation or deactivation of regulatory proteins [162]. 

All eukaryotic cells possess multiple MAPK pathways, and at least four major groups of MAPKs have been characterized in mammalian cells such as the c-Jun N-terminal kinases (JNK), the extracellular signal-related kinases (ERK1/2), the p38 kinase (p38), and the big MAP kinase 1 (BMK1/ERK5), which can be stimulated by cytokines, growth factors, and cellular stress even though their relative activation and the specific cellular response evoked depend on the different stimuli [163]. 

ERK, JNK, p38, and BMK1 are all proline-directed serine/threonine kinases, and the pathways in which they are activated also share similar homology. Indeed, all MAPKs are activated through a cascade of phosphorylation events, often referred to as the MAP kinase module, in which a MAP kinase kinase kinase (MAPKKK) phosphorylates and activates a MAP kinase kinase (MAPKK), which in turn phosphorylates and activates a MAPK [162,164].

A well-studied member of the MAPKKK family that is preferentially activated in response to various types of exogenous and endogenous cellular stress, including oxidative stress, is modulated by redox mechanisms, and mediates cell apoptosis, is apoptosis signal-regulating kinase-1 (ASK-1), a serine/threonine protein kinase that activates both p38 and JNK pathways [165]. In unstimulated conditions, ASK-1 binds to the repressor protein thioredoxin (Trx), a ubiquitously expressed redox regulatory protein, so that its kinase activity is inhibited. The binding of Trx to ASK-1 requires the presence of a reduced form of an intramolecular disulfide bridge between two cysteine residues in the catalytic site of Trx. This protein, after its oxidation by ROS molecules such as H_2_O_2_, dissociates from and liberates ASK-1, which is then activated by formation of an oligomeric complex and threonine autophosphorylation [165]. 

Several studies, which have revealed a direct link between ASK-1 and NOX, have also suggested that ASK-1 is an important effector of NOX in the redox signaling involved in cellular stress responses [166].

It has been shown that the three best-characterized MAPK subfamilies, JNK, p38 MAPK, and ERK, are activated by oxidative stress and could potentially be involved in pathways affecting the breakdown of muscle proteins or loss of nuclei via myonuclear apoptosis [160,167]. It has also been shown that H_2_O_2_ can elicit the activation of ERK, JNK, and p38 MAPK in skeletal myoblasts in a dose-and time-dependent manner [168]. On the other hand, it has been shown that exercise, as an intermittent form of cellular stress, is able to activate ERK1/2, p38, and JNK pathways in rat skeletal muscle [169]. 

Subsequent studies on the adaptations of muscle cells to exercise-linked oxidative stress have led to conclude that these distinct signaling pathways are partially dependent on the type, duration, and intensity of the contractile stimulus, and are critical cellular responses to maintain muscle homeostasis through upregulation of the expression of antioxidant enzymes and other cytoprotective proteins [170].

### 12.2. ERK 

ERK is composed of two isoforms, ERK1 and ERK2, collectively referred to as ERK1/2 [162,164]. Several mitogens, including epidermal growth factor, platelet-derived growth factor, and ROS can activate ERK1/2 [162] The activation of ERK1/2 by oxidative stress is consistent with the idea that low, but adequate levels of ROS are mitogenic [164]. Once activated, the ERKs can phosphorylate different substrates, including other kinases and transcription factors, and are involved in mediating different responses, that depend on which ERK substrates the cell expresses [171].

Activation of ERK1/2 regulates the transcriptional activity of activator protein-1 (AP-1) [172], avian myelocytomatosis virus oncogene cellular homolog (c-Myc) and the cell survival protein B-cell lymphoma-2 (Bcl-2) [173]. Although it can be considered an oversimplification, in general, ERK1/2 activation seems to promote cellular adaptations that lead to survival [164]. 

ERK1/2 is phosphorylated rapidly and transiently in response to mechanical stress. Indeed, it was demonstrated that ERK1/2 is activated in skeletal muscle of rats running on a motorized treadmill for 10–60 min [169] and in a rat plantaris in situ preparation stimulated to contract for 5 min by electrical stimulation [174].

Early human studies showed an increase in ERK1/2 phosphorylation after endurance-type exercises, including acute submaximal cycling [175] and marathon running exercise [176]. Subsequent works also showed an increase in ERK1/2 phosphorylation in response to resistance exercise [177,178].

The magnitude of ERK1/2 phosphorylation during endurance exercise correlates with the intensity of the protocol [175]. Conversely, resistance exercise upregulates ERK1/2 signaling in a manner that does not seem to preferentially depend on exercise intensity [179].

Furthermore, one-legged cycling exercise leads to an increase in ERK1/2 phosphorylation only in the exercised legs, suggesting that phosphorylation is dependent on local rather than systemic factors [174,180]. This view is supported by observation that ERK1/2 phosphorylation increases in isolated rat [181,182] and mouse [183] skeletal muscles stimulated to contract in vitro.

The effect of chronic training has been studied on rats subjected to a program of either low- or moderate-to-high-intensity treadmill running. ERK1/2 phosphorylation was similar to sedentary values, whereas ERK1/2 expression was increased three- to fourfold irrespective of the prior training program in muscle sampled 48 h after the last exercise bout [184]. 

Contrary to previous studies, chronic endurance training does not greatly influence total MAPK protein expression and pERK/total-ERK in chronically trained runners [185]. Furthermore, total ERK1/2 content was lower in powerlifting and weightlifting trained subjects compared to their controls [186].

### 12.3. p38

p38 is activated in response to various physiological stresses, such as osmotic stress, endotoxins and ROS [164]. ASK-1 represents the link between oxidative stress and p38 activation, because it is activated in response to ROS, such as H_2_O_2_, and is required for phosphorylation-mediated activation of both p38 and JNK [161]. Five isoforms of p38 have been identified (p38*α*, p38*β*, p38*β*2, p38*γ* and p38*δ*), whose expression differs in the various tissues, with p38*γ* predominantly expressed in skeletal muscle [162].

Among the phosphorylation targets of p38 there are several important transcription factors, including tumor protein p53, a phosphoprotein crucial in prevention of cancer formation [187], nuclear factor *κ*-light-chain-enhancer of activated B cells (NF-*κ*B), involved in the induction of antioxidant enzymes [188], and activating transcription factor 2 (ATF2), which regulates the transcription of various genes, including those involved in DNA damage response [189]. Of particular importance to apoptosis is the fact that activation of p53 results in the expression of the pro-apoptosis protein, bcl-2-like protein 4 (Bax), which can promote caspase-3 activation via a mitochondrion mediated pathway [190]. 

Although ERKs, JNKs and p38 are all activated by H_2_O_2_ treatment, following a time- and dose-dependent pattern in C2 skeletal myoblasts, the time-course of this activation differs among the MAPK subfamilies. Indeed, p38 activation is more rapid and displays a biphasic pattern, with a second peak obtained at 2 h of treatment [168]. This p38 re-activation could be attributed to a feedback mechanism, mediated by its either upstream activators or downstream targets, a phenomenon previously reported [163].

Phosphorylation of p38, like that of ERK1/2, increases during contraction of isolated skeletal muscles, implying a local activating factor [182,191]. Moreover, it is also increased by treadmill exercise in rodents [139] and cycling ergometry [174] and marathon running [176] in humans.

### 12.4. JNK

There are three isoforms of JNK (JNK1, JNK2, and JNK3) that are encoded by three different genes. JNK1 and JNK2 are ubiquitously expressed, while JNK3 is only expressed in brain, heart and testis [162]. Many of the stimuli that activate p38, including endotoxins, osmotic stress, and ROS, can also activate JNK. Moreover, like p38 activation, even activation of JNK induced by oxidative stress occurs via the ASK1 pathway [192]. The transcriptional factors AP-1, p53 and c-Myc and many other non-transcriptional factors, such as Bcl-2 family members, are among the specific molecular targets of JNK. About this, evidence has been obtained that JNK plays a major role in ROS-mediated apoptosis. Indeed, because ROS themselves are not able to activate caspases, JNK is required as another death-signaling pathway for oxidative stress-mediated apoptosis [161,192].

Signal transduction through the JNK pathway is also stimulated by intense exercise protocols and by those inducing muscular damage [193]. JNK phosphorylation increases linearly with increasing levels of muscular contraction force [174]. Therefore, JNK activity appears to be modulated by total muscle tension rather than duration of the contraction stimulus [194]. 

Summarizing, all three MAPK signaling pathways appear to be responsive to exercise even though their activation mechanisms (i.e., energetic/metabolic compared to mechanical) remain distinct. It is likely that the pattern of MAPK signaling have important implications in the different adaptive responses elicited by exercise. Indeed, MAPK may play an important role as a cellular intermediary able to couple perceived alteration in stress with adaptive changes, including the transcriptional regulation of redox state of the skeletal muscle. 

### 12.5. MAPK and Modulation of Gene Expression

In order to execute their functions, the MAPKs phosphorylate hundreds of substrates, thus modulating their activities. MAPK substrates were identified in the cytoplasm, mitochondria, Golgi apparatus, endoplasmic reticulum, and particularly the nucleus where they modulate gene expression [195,196,197]. Indeed, stress responses, as well as other cellular processes, are mediated by MAPK cascade-dependent induction and regulation of *de-novo* gene expression [198]. For this to happen, it is necessary that the signals transmitted via the various cascades enter the nucleus where they modulate the activity of transcription factors, transcription suppressors, and chromatin remodeling proteins, in order to ensure the correct cellular responses [199].

In fact, inside the cell nucleus, the DNA is packed into the chromatin, a structure consisting of protein–DNA complexes [200]. This structure is very compact so that it is not accessible to other proteins, including transcription factors. Therefore, the transcription requires a “decompaction” and a change into active open euchromatin. Following various types of stimulations, several distinct processes become operative to induce chromatin remodeling and allow the access to the target genes. Such processes include histone acetylation, histone phosphorylation, poly ADP ribosylation, changes in DNA conformation, and binding of other proteins to the DNA. Some of them are regulated by the cascades of MAPKs, including in particular ERK1/2 and p38s, and are required for the correct transcription and the induction of processes dependent on MAPKs [201]. At present, among the numerous substrates of MAPKs, several transcription factors have been identified. 

### 12.6. ROS Sensitive Transcription Factors

Over the past years, it was reported that MAPKs can regulate a wide range of transcription factors involved in response to oxidative stress elicited by exercise in skeletal muscle. On the other hand, based on the growing appreciation of the influence exerted by redox-sensitive signaling pathways on normal cellular processes, a reasonable hypothesis was that an important regulator of the adaptation in skeletal muscle in response to aerobic exercise may be ROS generated during the exercise.

ROS play a very important role to regulate several cell functions modulating the activity of preexisting proteins and inducing the expression of many genes via activation of specific redox-sensitive transcription factors [202]. Due to ROS involvement in almost all-important biological functions, it is difficult to define all the pathways and gene targets that redox signaling affects during exercise. Therefore, our examination will be limited to some of the most relevant factors that play critical roles in homeostatic regulation of muscle oxidant-antioxidant balance during exercise.

ROS are critical in the regulation of several transcription factors, including the activator protein-1 (AP-1) and the nuclear factor κ-light-chain-enhancer of activated B cells (NF-κB) [203,204,205] two transcription factors known to play crucial functions in proliferation, differentiation, and morphogenesis.

AP-1 and NF-kB response elements are located in the promoter regions of genes encoding CAT, GPX, Mn-SOD and CuZnSOD [204] and have been identified as the main factors that are both activated by exercise-produced ROS and directly implicated in the induction of the aforementioned antioxidant enzymes [206]. Moreover, combinations of AP-1 and NF-kB with other redox-sensitive transcription factors can determine which antioxidant enzyme is about to be induced and to what extent.

### 12.7. NF-κB

NF-κB, one of the most commonly investigated redox sensitive transcription factors, is a heterodimer composed of two related subunits, p65 and p50, which share a homologous region at the N-terminal end, necessary for DNA binding and dimerization.

The NF-κB/Rel transcription factors are normally sequestered in the cytoplasm in an inactive state, linked to the IκBα inhibitory protein. NF-κB is activated by several stimuli, including H_2_O_2_, proinflammatory cytokines, lipopolysaccharide (LPS), and phorbol esters, by the phosphorylation of IκBα at Ser-32 and -36 by IκB kinase (IKK). Phosphorylation of IκBα results in its dissociation from NF-κB and subsequent proteasomal degradation. NF-κB, once free, migrated into the nucleus where it binds to the corresponding DNA sequence of the target genes, including MnSOD and γ-glutamylcysteine synthetase (GCS) [207], the rate-limiting enzyme in the biosynthesis of glutathione.

In muscle cells, ROS such as H_2_O_2_ are able to induce degradation of the inhibitory IκB protein subunits bound to NF-κB subunits (p65, p50 and RelB), leading to the rapid migration of NF-kB to the nucleus and activation of the transcription of specific genes [203]. Zhou et al. [204] showed that the use of specific NF-κB inhibitors blocked the upregulation of the expression of Cat and Gpx induced by oxidative stress, thus confirming the hypothesis that ROS are able to modulate mRNA levels of antioxidant enzymes by activating redox-sensitive transcription factors, such as NF-κB.

In 1997, Sen et al. [208] were the first to bring NF-κB to the attention of exercise physiologists, demonstrating that NFκB activation in L6 muscle cells was responsive to H_2_O_2_ treatment and was controlled by intracellular GSH: GSSG status. Subsequently, Hollander et al. [134] reported that NF-κB (and AP-1) binding was significantly increased in rat skeletal muscle in a fiber-specific manner after an acute bout of prolonged exercise. Since NFκB binding was associated with an increase in MnSOD mRNA level and protein content, the authors hypothesized that NF-κB activation by ROS generated in contracting muscle may be the underlying mechanism for training adaptation and increase in antioxidant enzyme expression. Ji et al. [209], examining NFκB signaling cascades in response to exercise in rats, found that acute exercise increased NF-κB binding, IKK activity, IκBα phosphorylation and degradation, and P50 accumulation in the nucleus in rat DVL muscle. The exercise-induced activation of NF-κB was partially abolished by treatment with pyrrolidine dithiocarbamate, an inhibitor of the 26S proteosomes. Furthermore, the treatment with a high of t-butylhydroperoxide had scant effect on NF-κB, suggesting that the signaling was not induced by general oxidative stress but by specific chemical agents. 

In a subsequent study, Ho et al. [210] found an increase in NF-κB activation accompanied by IKKα/β phosphorylation in the rat soleus (type 1) and red gastrocnemius (type 2a) muscles during 60 min of treadmill exercise. Peak IKKα/β activation was found early during exercise (15 min), whereas maximal NF-κB binding was found at 1–3 h. IKKα/β and IκB phosphorylation was also increased by the contraction of isolated extensor digitorum longus (EDL) muscles in vitro. Moreover, application of p38 and ERK inhibitors reduced IKKα/β activation, suggesting that MAPKs were upstream of NF-κB and could partially mediate stimulation of NF-κB activity by contraction.

Gomez-Cabrera et al. [53] found that an acute bout of treadmill running in rats activated ERK1/2 and p38 and the activation coincided with elevated gene expression of MnSOD and iNOS. Moreover, when an inhibitor a xanthine oxidase (XO), allopurinol, was used to partially block ROS generation, MnSOD and iNOS mRNA expression induced by exercise was severely hampered, and the activities of ERK, p38, and NFκB were decreased. Although it was not possible to conclude that attenuation of MAPK signaling was the reason for the decreased MnSOD and iNOS expression, these results suggested that MAPK proteins played a role in the signaling of antioxidant enzymes and that an integrated input from both the NFkB and MAPK signaling pathways was required to stimulate gene expression of these enzymes in the muscle fibers. These results also suggested that nonmitochondrial ROS were involved in the improvement of muscle antioxidant defense system.

### 12.8. AP-1

AP-1 is a heterodimer consisting of activating (c-Fos and c-Jun) and inhibitory (Fos-related antigen (Fra)-1 and 2) subunits, which can generate different heterodimers, thus modulating expression of target genes [211]. Depending on the cell type and cellular redox milieu, Fos and Jun can dimerize or interact with other transcription factors such as activating transcription factor (ATF), CCAAT enhancer binding protein (C/EBP), and proto-oncogene (Maf) leading to either activation or inhibition of gene transcription of antioxidant and immunoactive proteins [212,213].

AP-l regulates the gene expression in response to signals generated by a wide variety of extracellular stimuli, among which growth factors, tumor promoters, neurotransmitters, UV light, and cytokines [214,215]. AP-1 can also be activated by ROS [216] and oxidative stress induces the binding of AP-1 complex proteins (c-Jun and c-Fos) to DNA [211]. According to this observation, an increase in AP-1 binding has also been found after a single bout of exercise [134].

Activation of various kinases which are involved in the MAPK signaling pathway can lead to the sequential phosphorylation of a variety of proteins, resulting in increased expression of c-Jun, a subunit of the transcription factor AP-1, which is an important DNA-binding site on many genes able to respond to oxidative stress [217].

It has been reported that c-Jun is regulated by JNKs to which it gave the name [218]. c-Jun is constantly expressed in both unstimulated and stimulated cells. Upon stimulation, c-Jun, to exert its activity, interacts with other transcription factors such as c-Fos, and ATF forming AP-1 [219]. The activation of c-Jun depends on phosphorylation of its transactivation domain by all JNK isoforms and to some extent by other MAPKs, which leads to induction of the full transcriptional activity within the AP-1 complex, independent of DNA binding [220].

### 12.9. Nrf2

Although the protection provided by NF-κB and AP-1 activation is important for cellular redox homeostasis, another pathway is the main regulator of cytoprotective responses to endogenous and exogenous stresses caused by electrophilic compounds and ROS [221]. The key signaling protein within the pathway is the transcription factor nuclear factor erythroid 2-related factor 2 (Nrf2) that can bind, together with small musculoaponeurotic fibrosarcoma (Maf) proteins, to a DNA sequence called antioxidant response element (ARE) in the regulatory regions of target genes. Nrf2 can also bind to Kelch ECH associating protein 1 (Keap1), a repressor protein very rich in cysteine residues most of which can be modified in vitro by different oxidants and electrophiles [222,223].

**Figure 2:**
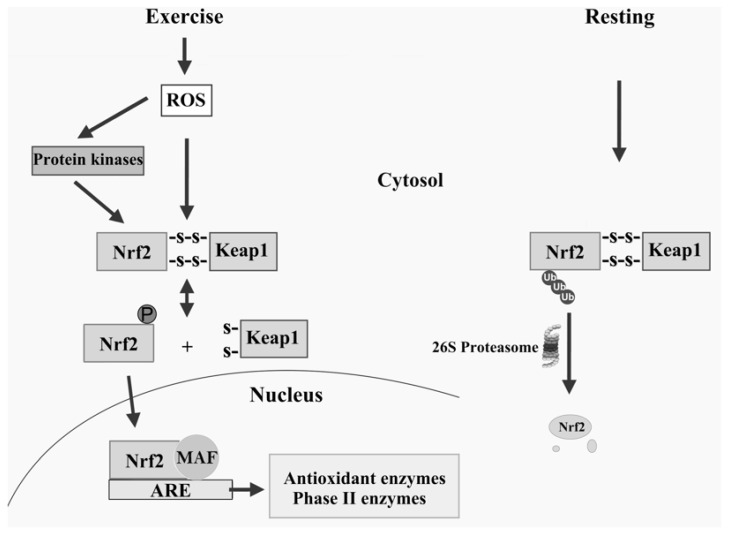
Schematic model of transcriptional activity of nuclear factor erythroid 2-related factor 2 (Nrf2) mediates by oxidants during exercise and Nrf2 degradation in resting condition. MAF, musculoaponeurotic fibrosarcoma protein; ARE, antioxidant response element; Keap1, Kelch ECH associating protein 1; Ub, ubiquitin.

In unstressed conditions, the cellular concentration of Nrf2 protein is maintained at very low levels by its inhibitor Keap1, which sequesters Nrf2 in the cytosol and facilitates its ubiquitination through the Keap1/Cul3 ubiquitin ligase and rapid proteasomal degradation. Under conditions of stress or in the presence of Nrf2 activating compounds, this degradation is hampered because modification of reactive cysteine thiols of Keap1 and Nrf2 by inducers presumably alters the structure of the Nrf2/Keap1/Cul3 complex, leading to inhibition of Nrf2 ubiquitination Nrf2 release. Subsequently Nrf2, phosphorylated by protein kinases, moves into the nucleus where it forms heterodimers with Maf proteins. This, in turn, facilitates the binding of Nrf2 to the antioxidant response element (ARE), a cis-acting enhancer sequence (TCAG/CXXXGC) in the promoter region of Nrf2-regulated genes [224,225] (Figure 2).

Genome-wide search for Nrf2 target genes has led to identify an array of ARE-regulated genes, that lead to the production of phase II xenobiotic metabolizing enzymes, antioxidants, molecular chaperones, DNA repair enzymes, and anti-inflammatory response proteins [226]. They reduce reactive compounds such as electrophiles and free radicals to less toxic intermediates and increase cell capacity to repair any damage ensued.

An alternative model of the Nrf2-Keap1 pathway of gene regulation has also been proposed [227]. According to this model, Nrf2 is constitutively expressed in cells and moves directly into the nucleus to activate gene transcription. Nrf2 is then targeted for degradation by Keap1, a process that requires the transient Keap1 displacement in the nucleus. In cells under stress, the stabilization of Nrf2 in response to activating compounds is caused by mechanisms that prevent Nrf2 from binding to Keap1 and being degraded in the nucleus. The reduced degradation of Nrf2, together with its de novo synthesis, results in the accumulation and direct recruitment of Nrf2 to the ARE, so that transcription of its genes increases. This pathway of regulation of Nrf2 activity should allow it to exert its dual function of controlling gene expression constitutively and inducibly.

Whatever the pathway of gene regulation may be, the transcription factor Nrf2 is certainly the master regulator of cellular antioxidant defense, because it regulates more than 200 cytoprotective genes in response to oxidative stress [228]. Nrf2 can regulate many antioxidative enzymes, including haem oxygenase-1, SOD, CAT, and NADPH quinone oxidoreductase [229]. Nrf2 also make sure that the antioxidant enzyme expression is coupled with the cofactor supply. Indeed, it controls the expression of GPX2 [230], which reduces peroxides producing GSSG, and GR1 [230], which reduces GSSG, thus allowing intracellular levels of GSH to remain constant. In addition to the GSH-based antioxidant system, Nrf2 also controls the expression of cytosolic Trx1 [231] TrxR1 [230,232] and sulfiredoxin (Srx1) (a cysteine sulfinic acid reductase) [233], all of which reduce oxidized protein thiols [234]. Antioxidant enzymes, such as GR1 and TrxR1, require NADPH as a cofactor so that it is notable that NADPH-generating enzymes such as glucose- 6-phosphate dehydrogenase, 6-phosphogluconate dehydrogenase, isocitrate dehydrogenase, and malic enzyme are all regulated by Nrf2 [235]. 

Interestingly, Nrf2 also contributes to the maintenance of metabolic homeostasis since Nrf2 induction in pancreatic β cells markedly suppresses oxidative-stress-mediated dysfunction [236].

Evidence is available that the Nrf2 pathway also plays a key role in how oxidative stress mediates the exercise beneficial effects. Increases in ROS production induced by bouts of acute exercise stimulate Nrf2 activation and when they are applied repeatedly, as with regular physical activity, this may lead to upregulation of endogenous antioxidant defenses and overall greater capacity to counteract the oxidative damage of biological molecules. 

Cell culture study using C2C12 skeletal muscle cells provided evidence that Nrf2 is activated by ROS and this activation is suppressed when antioxidants, such as N-acetylcysteine, are added to the culture medium [237]. Subsequent study showed an increase in Nrf2 protein expression after myotube treatment of myotubes with H_2_O_2_ [238].

A single bout of acute exercise in wild-type mice has been shown to increase Nrf2 gene expression [238,239], Nrf2 protein abundance in skeletal muscle [239], and Nrf2-dependent phase II enzymes [238,240]. Conversely, no change in Nrf2 activity was observed in Nrf2^–/^^–^ mice after acute bout of exercise [239]. 

The exercise increased oxidative stress and activated Ref1/Nrf2 signaling in a time-dependent manner, with a linear correlation between the mitochondrial H_2_O_2_ content and Ref1/Nrf2 expressions.

The effect of regular exercise training on the Nrf2 response has been studied extensively and it has been found that, regardless of duration or training regimen, regular aerobic exercise in rodent models activates Nrf2 signaling across multiple tissues including skeletal [241] and cardiac muscle [242,243].

Taken together, the studies demonstrate that regular exercise upregulates Nrf2 protein abundance and phase II and antioxidant enzyme amounts. Furthermore, emerging evidence suggests that an active lifestyle can conserve skeletal muscle cellular redox status via activation of Nrf2 –Keap1 signaling in elderly. Indeed, a cross-sectional study comparing Nrf2 and Keap1 protein content from a single muscle biopsy in sedentary and active older adults has shown the age-associated decline in antioxidant response is due, at least in part, to dysfunction in Nrf2–Keap1 redox signaling, which is preserved in the skeletal muscle of older adults thus maintaining cellular redox homeostasis [244]. However, it is not known whether an exercise program can restore redox balance in individuals who already display a Nrf2 signaling impairment, even though moderate exercise training has been shown to be able to restore Nrf2 signaling in cardiac muscle in older age [243].

### 12.10. PGC-1α

Although the molecular mechanisms of the adaptive response to exercise remain to be fully elucidated, PGC-1α, a transcriptional coactivator, is currently considered a major regulator of phenotypic adaptation induced by exercise. 

PGC-1α was first identified as a transcriptional coactivator of the peroxisome proliferator-activated receptor (PPAR)-γ in brown fat cells [245]. Subsequently, it was found in other mitochondria-rich tissues, including skeletal and cardiac muscle, as well as in kidney, liver, and brain [246] in which it influences numerous aspects of metabolism [247]. PGC-1α and its homolog PGC-1β are also co-activators for PPARα and PPARδ (involved in adipocyte differentiation and thermogenesis), and for a variety of other transcription factors [248,249]. Furthermore, PGC-1α promotes upregulation of itself by an interaction with myocyte enhancer factor 2 (MEF2) on its own promoter [248].

**Figure 3:**
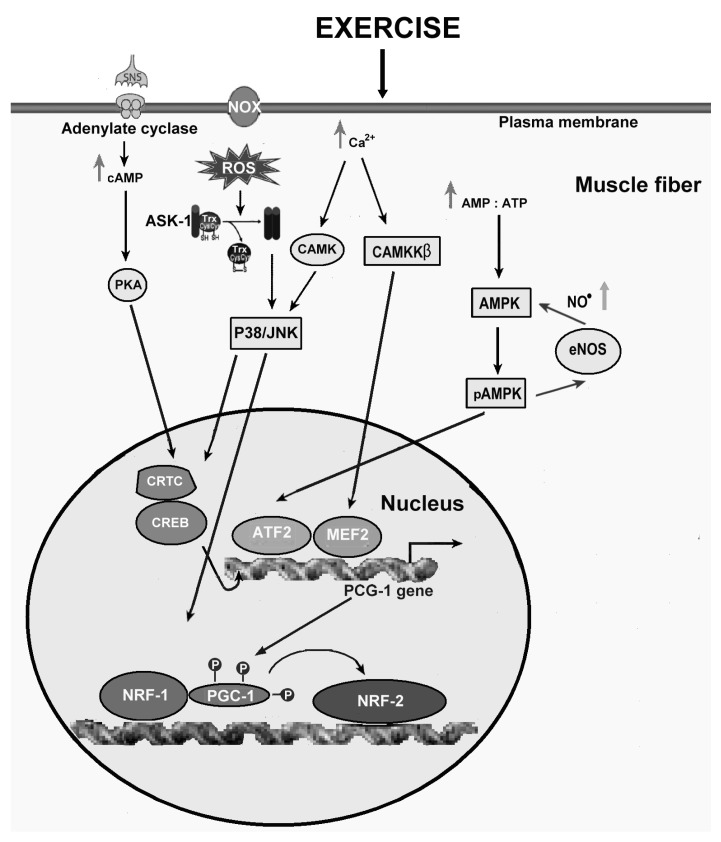
Schematic representation of the signalling pathways that mediate the exercise- induced PGC-1 expression and mitochondrial biogenesis in skeletal muscle. PGC-1, peroxisome proliferator–activated receptor coactivator 1; NRF-1, nuclear respiratory factor 1; NRF-2, nuclear respiratory factor 2; ATF2, activating transcription factor 2; MEF2, myocyte enhancer factor-2; cAMP, cyclic adenosine monophosphate; CREB, cAMP response element-binding protein; CRTC, cAMP-regulated transcriptional co-activators; AMPK, AMP-activated protein kinase; PKA, protein kinase A; NO^•^, nitric oxide; eNOS, endothelial nitric oxide synthase; CAMK, Ca2+/calmodulin-dependent protein kinase; p38, p38 mitogen-activated protein kinases; JNK, c-Jun N-terminal kinase; ASK-1, apoptosis signal-regulating kinase-1.

PGC-1α can interact with nuclear receptors and transcription factors activating transcription of their target genes, and its activity is responsive to a wide variety of stimuli including calcium ion, ROS, insulin, thyroid and estrogen hormones, hypoxia, ATP demand, and cytokines [249] (Figure 3).

For a long time PGC-1α has been considered to be exclusively a master regulator of mitochondrial biogenesis by coactivating numerous transcription factors that, in turn, bind to the promoters of distinct sets of nuclear-encoded mitochondrial genes [250]. However, more recent studies have shown that PGC-1α is also able to stimulate the expression of endogenous antioxidant proteins. Reduced mRNA levels of CuZnSod, MnSod, and/or Gpx1 [251], as well as MnSOD protein content [252,253], were found in skeletal muscle from PGC-1α knockout mice compared to wild type, while PGC-1α overexpressing mice showed an increase in MnSOD protein content [254]. PGC-1α KO fibroblasts exhibited a decrease in MnSod, Cat, and Gpx1 mRNA content relative to wild-type fibroblasts and PGC-1α KO mice were more vulnerable to oxidative stress [255]. Furthermore, PGC-1α is able to regulate RNA expression of UCP2 and UCP3 in cell culture [150], suggesting that PGC-1α may increase the uncoupling capacity of mitochondria, thus reducing their ROS production. PGC-1α also promotes mSIRT3 gene expression, which is mediated by an ER-α binding element mapped to the SIRT3 promoter region [256]. SIRT3, in turn, binds to mitochondrial enzymes, including MnSOD, and activates them by deacylation [257,258]. Taken together, PGC-1 α appears to play a role in reducing cell oxidative damage by upregulating antioxidant gene expression and activity. 

Interestingly, recent report has shown that PGC-1 is also necessary for the activation of the signaling network called unfolded protein responses (UPR) during pharmacologically induced endoplasmic reticulum stress and exercise training [259].

PGC-1 protein expression increases rapidly in muscle fibers stimulated to contract [260]. Moreover, Pgc-1 gene expression increases in rat skeletal muscle after a single bout of exercise [261] and in human skeletal muscle after endurance training [262]. Increased levels of PGC-1α protein expression were also found in rat skeletal muscle after 10 weeks of training to swimming [263].

It is worth noting that several initiating stimuli, activated during exercise, can contribute to induction of the PGC-1 gene response. First, acute exercise leads to rapid activation of p38 [264], which in turn activates PGC-1α by phosphorylation [265] and produces the increase in its expression [266].

Other stimuli activated by exercise, that are able to induce Pgc-1 gene response include: (i) Increased concentration of cytosolic calcium, which activates several signaling pathways regulated by the calcineurin phosphatase and the calmodulin-modulated kinase, (ii) decreased levels of high-energy phosphates, which lead to AMPK activation of, (iii) stimulation of the adrenergic system, which leads to cyclic AMP synthesis, and activation of various kinases, including protein kinase A [151] (Figure 3). 

However, it is worth noting that the regulation of PGC-1α is not limited to variations in its expression but is also dependent on covalent modifications including phosphorylation, acetylation, methylation and ubiquitination [267]. Indeed, in vitro experiments have shown that PGC-1α phosphorylation by p38 MAPK and AMPK produces a more active protein [151].

Most studies point toward H_2_O_2_ as an important molecule for PGC-1α upregulation in skeletal muscle. ROS involvement in contraction-induced increases in Pgc-1α expression is supported by the observation that the increase in Pgc-1α mRNA, induced by electrical stimulation in cell culture of rat skeletal muscle, is prevented by antioxidant incubation [268]. Thus, the idea that the upregulation of ROS-removing enzymes in response to increases in ROS can be in part mediated by PGC-1α is supported by the observation that the increase in the Sod, Cat and Gpx mRNA content induced by H_2_O_2_ in Pgc-1α KO fibroblasts is lower than that in wild-type fibroblasts [255].

Furthermore, the observation that treatment of cultured muscle myotubes with exogenous H_2_O_2_ activates AMPK and increases Pgc-1α expression [268] suggests that H_2_O_2_ can promote Pgc-1α expression through AMPK. Moreover, the sensitivity of PGC-1*α* to the redox status is confirmed by the observation that the antioxidant *N*-acetylcysteine inhibits Pgc-1α upregulation [269].

Pharmacological inhibition of xanthine oxidase with allopurinol also suppresses the upregulation of PGC-1α induced by a single bout of anaerobic exercise in parallel to blunted activation (i.e., phosphorylation) of p38 MAPK in rat vastus lateralis muscle [75]. This finding suggests that ROS generated in response to in vivo contraction are involved in p38 MAPK activation and subsequent regulation of PGC-1α expression [75]. Moreover, evidence that allopurinol treatment also reduces the exercise-induced increases in levels of transcription factors, such as nuclear respiratory factor 1 (NRF-1) and factor of transcription A (Tfam), which are involved in mitochondrial biogenesis, indicates that ROS arising from nonmitochondrial sources play a major role in stimulating mitochondrial biogenesis [75]. 

Although in literature there are conflicting results [270], ROS have also been shown to be functionally important for PGC-1α expression and adaptive responses induced by endurance exercise in skeletal muscle. Indeed, several experimental studies have reported that antioxidant supplementation attenuates the increase in Pgc-1 gene expression [271,272] and PGC-1 protein content [263,273] elicited by endurance training. 

Antioxidant supplementation also prevent health-promoting effects of physical exercise, including mitochondrial biogenesis [263,271,273], endurance performance (running to exhaustion) [273], and insulin sensitivity [271]. These results strongly support the view that the ROS generated during each session of exercise can cause beneficial effects functioning as signals regulating molecular events critical for muscle adaptive responses to training.

It is worth noting that PGC-1*α* expression in muscle can be regulated by a variety of stimuli associated with muscular exercise, which, however, seem to be dependent on ROS production. Thus, the finding that the human PGC-1*α* promoter contains a binding site for NF-*κ*B suggests that the expression of PGC-1*α* may also be regulated by NF-*κ*B [274]. Analysis of the human PGC-1*α* promoter has revealed a variety of consensus transcription binding sites to the following transcription factors: Specificity protein 1 (SP1), cAMP response element binding protein (CREB), CREB related family member, activating transcription factor 2 (ATF2), forkhead transcription factor (FKHR), p53, EBox binding proteins, GATA and muscle enhancer factor 2 (MEF2) [274]. Many of these transcription factors have been shown to be ROS-sensitive, which indicates numerous potential possibilities for redox control of PGC-1*α* expression.

Additionally, RNS, particularly NO^•^, may also be involved in the regulation of PGC-1*α*. The idea that NO^•^ mediates the upregulation of PGC-1a, thus modulating mitochondrial function and biogenesis, is supported by the evidence that low levels of NO^•^ induce mitochondrial biogenesis, PGC-1a and GLUT4 expression in cultured muscle cells [275] and that a genetic deletion of NOS or their pharmacological inhibition prevents PGC-1a induction that is triggered by endurance exercise [276]. 

It has also been observed that administration to humans of inorganic nitrate (which can be converted into NO^•^ in the body) significantly improves energy metabolism during exercise [277]. Reports showing that NOS activity is involved in mitochondrial biogenesis induced by AMPK and CaMK and PGC-1α expression in L6 myotubes [278] and that AMPK phosphorylates and activates both eNOS and nNOS [279], led to propose that there is a positive feedback loop between NO^•^ production and AMPK activity in skeletal muscle [280]. The evidence that NO^•^ production promotes PGC-1*α* expression via NO-mediated activation of AMPK (i.e., AMPK*α*1 isoform) demonstrated that the proposed model of synergistic interaction between AMPK and NOS is crucial to maintain metabolic function in skeletal muscle cells [280]. Moreover, it suggested that both ROS and RNS can contribute to PGC-1*α* expression via a common signaling pathway (i.e., AMPK activation). 

## 13. Regulation of Cellular Phosphatases by ROS

It has been previously pointed out that changes in the phosphorylation status of signaling molecules play an important role in the control of cellular adaptation. In this regard, it is necessary to note that the phosphorylation status of regulatory proteins and/or transcriptional activators is regulated not only by kinase activity but also by changes in phosphatase activity.

In general, phosphatases are divided into two major classes (i.e., serine/threonine phosphatases and phosphotyrosine phosphatases), both of which are known to be redox sensitive in many different cell types, including skeletal muscle. Serine/threonine phosphatases contain metal ions that are susceptible to oxidation, which leads to their inactivation. Similarly, the phosphotyrosine phosphatases (PTPs) are susceptible to oxidation-induced inactivation. The PTPs contain a cysteine residue in their active site, and oxidation of this cysteine inactivates the enzyme [281]. A subclass of PTPs, called dual-specificity phosphatases (DUSPs), can remove phosphates from both tyrosine and serine/threonine residues. The DUSPs contain two cysteines in their active sites, leading to inactivation of the enzyme during oxidizing conditions. DUSP family include 10 members, which differ in their substrate specificity, subcellular localization, tissue expression, and inducibility by extracellular stimuli [282].

MAPKs including ERK1/2 are dephosphorylated on both the threonine/serine and tyrosine residues by MAPK phosphatases (MKPs) belonging to the DUSP family. It has been shown that, in human skeletal muscle, ERK1/2 phosphorylation is increased in an intensity-dependent manner by acute contractions, but after exercise this phosphorylation is rapidly reduced, and resting levels are restored within 60 min [175,283]. Recent study has demonstrated that two ERK1/2-specific MKPs, dual specificity phosphatase 5 (DUSP5) and DUSP6, are the most regulated MKPs in skeletal muscle after acute exercise [284]. DUSP5 expression is nine-fold higher immediately after exercise and returns to pre-exercise level within 2 h, whereas DUSP6 expression is reduced by 43% just after exercise and remains below pre-exercise level after 2 h recovery. It has also been proposed a hypothetical interplay between ERK1/2 signaling, DUSP5, and DUSP6 in skeletal muscle before and after exercise. Before exercise, basal phosphorylation of MEK (the kinase phosphorylating ERK1/2) and ERK1/2 is low and inactive ERK1/2 is bound to inactive MEK and DUSP6 in cytoplasm. During exercise MEK is activated leading to increased phosphorylation and translocation into the nucleus of ERK1/2, which enhances expression of its target genes, including DUSP5, which, in turn, increases dephosphorylation and trapping of ERK1/2 in the nucleus and reduces ERK1/2 recycling to cytoplasm. A higher proportion of cytoplasmic ERK1/2 is available for phosphorylation by MEK due to the reduced level of DUSP6. During recovery, MEK and ERK1/2 activities are reduced to the basal level, normalizing the DUSP5 level. ERK1/2 is translocated back to the cytoplasm and most of it is bound to MEK, whereas DUSP6 level is still low [284].

## 14. Conclusions 

The idea is now widely shared that the utilization of oxygen by aerobic organisms exposes them to the attack of reactive oxygen and nitrogen species, which can initiate chain reactions leading to oxidative damage of important biological molecules. Aerobic organisms are provided with an efficient antioxidant defense system that allows them to neutralize the oxidative effects of reactive metabolites of oxygen and nitrogen. However, when reactive species production exceeds the cellular antioxidant capacity, oxidative stress develops, potentially leading to cell structural and functional alterations and to the development of many pathological conditions. A single session of strenuous or prolonged exercise leads to the production of high number of radicals and other reactive oxygen species (ROS), which cause tissue damage and dysfunction. On the other hand, regular exercise appears to decrease the incidence of a wide range of ROS-associated diseases because the single sessions of a training program produce low amounts of ROS, which can induce adaptive responses beneficial for the organism. Cells may adapt to the stress by upregulation of systems of defense against and repair of oxidative damage so that they are then resistant to higher levels of oxidative stress imposed subsequently. 

Although the effects of exercise on antioxidant capabilities in skeletal muscle have been well described, the regulatory mechanisms underlying this adaptation are complex and incompletely understood. To date it is known that cells exposed to ROS are brought to respond by inducing or repressing a remarkable variety of target genes [155]. These effects appear to be due to modification of the intracellular redox balance resulting in the activation of several signaling pathways ultimately leading to a modulation of gene expression. A comprehensive answer to the question as to how changes in the redox status of muscle fibers regulate signaling pathways and gene expression is not yet available. However, it is clear that a major mechanism by which redox signaling is able to alter gene expression and modify muscle phenotype involves changes in phosphorylation status of transcriptional activators due to ROS capacity to control the activities of many kinases and phosphatases. Evidence that ROS play a pivotal role in adaptive response elicited by exercise training is provided by researches indicating that antioxidant supplementation blunts benefits of regular physical activity on skeletal muscle. 

In conclusion, ROS generated during muscle activity, from both mitochondrial and nonmitochondrial sources, may play a pivotal role in muscle adaptive responses to exercise-induced oxidative stress by activating redox-sensitive signal transduction. Important signaling pathways that can be activated in response to ROS stimulation include NFκB, Nrf2, and MAPK. Moreover, the existence of multiple redox-sensitive binding sites on antioxidant genes suggests that the fidelity of gene expression requires the synergistic activation and interaction of several transcription factors. These regulatory mechanisms may control not only the effectiveness of antioxidant defense system through upregulation of antioxidant enzyme expression but also other biological activities in skeletal muscle, including mitochondrial biogenesis.

## Figures and Tables

**Table 1:** Effect of exercise on markers of oxidative damage in skeletal muscle.

Species	Activity	Marker	Ref.
Rat (6 mo)	Exhaustive treadmill running (submaximal work intensity) (gastrocnemious, soleus, plantaris)	TBARS↑	[33]
Rat (2 mo	Exhaustive swimming (gastrocnemious)	HPs↑, MDA↑	[8]
Rat (12 mo)	Exhaustive swimming (gastrocnemious)	HPs↑, MDA↑	[9]
Rat (4 mo)	Acute swimming (6 h) (gastrocnemious)	HPs↑, MDA↑, C=O↑, GSH/GSSG↓, C=O (mit)↑	[50]
Rat	Moderate and high intensity running (red and white VL)	HPs↔, MDA ↑	[59]
Rat	Treadmill running (20 min)	MDA↑	[60]
Rat	Treadmill running (1 h) (20 m/min, O% grade)	MDA (mit)↑	[61]
Rat	Exhaustive exercise (gastrocnemious)	C=O↑	[64]
Rat (8 mo, 24 mo)	Exhaustive treadmill running (25 m/min, 15 m/min, 5% grade)	MDA↑, C=O↔, GSH/GSSG↓	[38]
Rat (2 mo)	Exhaustive treadmill running (1.6 Km/h) (fast and slow muscle)	C=O↔, MDA↔, 8-oxodG↔	[65]
Dog	Treadmill running (7 h) (splenius, diaphragm, gastrocnemious)	8-oxodG↔	[66]
Men (~26, ~65 yr)	Exhaustive treadmill running (45 min, 75%VO_2max_ and 45 min, 90% VO_2max_)	8-oxodG↑	[67]
Rat	Exhaustive treadmill running	GSH/GSSG↓	[72]
Men (~68 yr)	Whole-body resistance exercise training (14 wk)	8-oxodG↓	[124]
Rat (2 mo)	Swim training (10 wk) (gastrocnemious)	MDA↔	[8]
Rat (12 mo)	Swim training (10 wk) (gastrocnemious)	MDA↔	[9]

↔ unchanged; ↓reduced; ↑increased; mo: months; yr: years; wk: weeks; mit: mitochondria; VL: vastus lateralis.

**Table 2:** Effect of training on antioxidant enzyme activity in skeletal muscle.

Species	Activity	Enzymes	Ref.
Rat (2 mo)	Swim training (1 h, 10 wk) (gastrocnemious)	GPX↑, GR↑	[8]
Rat (12 mo)	Swim training (1 h, 10 wk) (gastrocnemious)	GPX↑, GR↑	[9]
Rat (50 days)	Swim training (1 h, 10 wk) (gastrocnemious)	GPX↑, GR↑	[117]
Mouse (2 mo)	Swim Training (1 h, 6 wk)	GPX↑, GR↑, MnSOD↔, CuZnSOD↑	[120]
Mouse (26 mo)	Swim training (1 h, 6 wk)	MnSOD↔, CuZnSOD↔	[120]
Rat	Treadmill training (32 m/min, 8%, 2 h, 12 wk) (soleus, gastrocnemious)	CAT↓, GPX↑, SOD ↔	[116]
Rat (2 mo)	Treadmill training (1 h, 13 wk, 50–60% of maximal exercise capacity) (soleus)	CAT↑, GPX↑, MnSOD↑, CuZnSOD↔	[119]
Rat (21 mo)	Treadmill training (1 h, 13 wk, 50–60% of maximal exercise capacity) (soleus)	CAT↔, GPX↔, MnSOD↓, CuZnSOD↔	[119]
Rat	Treadmill training (25 m/min 10%, 2 h, 10 wk) (DVL)	SOD↑, GPX↑, GR↓	[122]
Rat	Treadmill training (25 m/min 10%, 2 h, 10 wk) (soleus)	SOD↔, GPX↔ GR↓	[122]
Rat (4 mo)	Treadmill training (25 m/min, 10%, 10 wk) (DVL)	GPX↑, MnSOD↔, CuZnSOD ↑	[126]
Rat (3 mo)	Treadmill training (27 m/min, 12% grade, 2 h, 10 wk) (SVL, soleus, plantaris)	GPX↔ CAT↔, MnSOD↔, CuZnSOD↔	[127]
Rat (3 mo)	Treadmill training (27 m/min, 12% grade, 2 h, 10 wk) (DVL)	GPX↑CAT↑, MnSOD↑, CuZnSOD↔	[127]
Men (~23 yr)	Maximal cycling sprint training (6 wk) (VL)	GPX↔ GR↔, SOD↔	[54]
Men (~23 yr)	Maximal cycling sprint training (7 wk) (VL)	GPX↑, GR↑, SOD↔	[54]
Men (~71 yr)	Unilateral resistance exercise training (12 wk) (VL)	CuZnSOD↑ MnSOD↔ CAT↑	[125]

↔ unchanged; ↓reduced; ↑increased; mo: months; yr: years; wk: weeks; DVL: deep vastus lateralis; VL: vastus lateralis.
